# Telemedicine in the Management of Arterial Hypertension in Rural Populations: A Narrative Review

**DOI:** 10.3390/healthcare14101383

**Published:** 2026-05-18

**Authors:** Ainur Bilmakhanbetova, Serik Ibraev, Assiya Turgambayeva, Gulnara Kulkayeva, Telman Seisembekov

**Affiliations:** 1 Department of Public Health and Management, NJSC “Astana Medical University”, Astana 010000, Kazakhstan; serik_ibraev@mail.ru (S.I.); assiya739@gmail.com (A.T.); 2 National Research Oncology Center, Astana 010000, Kazakhstan; 3 National Research Centre for Healthcare Development Named After Salidat Kairbekova, Astana 010000, Kazakhstan; gulnara1412@mail.ru; 4 Heart Rhythm Research Institute, NJSC “Astana Medical University”, Astana 010000, Kazakhstan; seisembekov@mail.ru

**Keywords:** arterial hypertension, telemedicine, rural health, digital health, primary healthcare, mHealth, blood pressure control, healthcare access, teleconsultation

## Abstract

**Background:** Arterial hypertension is one of the most prevalent chronic non-communicable diseases and a leading cause of cardiovascular morbidity and mortality worldwide. Its burden remains particularly high in rural and resource-limited settings, where access to healthcare is often constrained by shortages of healthcare professionals, geographical barriers, and underdeveloped infrastructure. These factors may contribute to delayed diagnosis, suboptimal disease control, and increased risk of complications. In this context, telemedicine has emerged as a useful approach to supporting hypertension management and improving access to care in rural populations. **Methods:** This study presents a narrative review of the literature focusing on the application of telemedicine in the management of arterial hypertension in rural populations. A structured literature search of PubMed, Scopus, and Web of Science databases was conducted for studies published between 2015 and 2025. The review included randomized controlled trials, systematic reviews, meta-analyses, and observational studies evaluating telemedicine interventions, including remote blood pressure monitoring, mobile health applications, and teleconsultations. Study selection was guided by relevance to the research objective, with particular attention to rural and resource-limited contexts. **Results:** Telemedicine interventions have been associated with improvements in blood pressure control, treatment adherence, and access to healthcare services. Evidence from randomized controlled trials and meta-analyses suggests modest reductions in systolic and diastolic blood pressure compared with standard care. However, a substantial proportion of the available evidence originates from studies conducted in general or mixed populations rather than exclusively rural settings. Therefore, the applicability of these findings to rural contexts remains limited and should be interpreted with caution. The effectiveness of telemedicine may vary depending on differences in healthcare infrastructure, resource availability, digital accessibility, and organizational models across healthcare systems. Integrated care approaches involving primary healthcare providers and specialist support may contribute to improved continuity of care, although their impact appears to be context-dependent. Key barriers include limited telecommunication infrastructure, digital literacy challenges, and difficulties in integrating telemedicine into routine clinical practice. **Conclusions:** Telemedicine may represent a useful approach to supporting hypertension management in rural populations. However, its implementation requires careful consideration of local healthcare systems, patient characteristics, and organizational context. Telemedicine should be viewed as a context-dependent strategy rather than a uniform solution. Further context-specific research is needed to evaluate the long-term clinical, organizational, and economic impact of telemedicine interventions in rural hypertension management.

## 1. Introduction

Arterial hypertension remains a major global public health challenge and a leading modifiable risk factor for cardiovascular diseases, including stroke, myocardial infarction, heart failure, and chronic kidney disease. According to recent global estimates, elevated blood pressure contributes substantially to premature mortality and disability worldwide [1]. Despite the availability of effective pharmacological and non-pharmacological treatment strategies, optimal blood pressure control remains suboptimal, particularly in low- and middle-income countries and rural populations [2,3].

Rural healthcare systems face multiple structural and organizational challenges that significantly limit effective hypertension management. These include reduced access to healthcare facilities, shortages of qualified healthcare professionals, insufficient diagnostic resources, and geographical barriers that delay timely diagnosis and follow-up care. As a result, patients living in rural areas are more likely to have uncontrolled hypertension, delayed treatment initiation, and a higher risk of cardiovascular complications [3,4]. In addition, low levels of health literacy and limited access to continuous medical supervision further complicate long-term disease management. These challenges may also contribute to differences in care complexity and clinical trajectories in rural populations.

In recent years, telemedicine has emerged as a promising approach to overcoming these barriers by enabling the remote delivery of healthcare services through digital technologies. Telemedicine encompasses a wide range of interventions, including remote blood pressure monitoring, mobile health (mHealth) applications, teleconsultations, and digital decision-support systems. These technologies facilitate continuous patient monitoring and may improve access to specialist care and enhance communication between patients and healthcare providers [5,6,7].

In the context of arterial hypertension, telemedicine interventions have shown potential to improve clinical outcomes. Evidence from randomized controlled trials and meta-analyses suggests that telemedicine may be associated with improvements in blood pressure control, treatment adherence, and patient engagement compared to standard care [8,9,10,11,12,13,14,15,16].

In addition, it should be noted that a substantial proportion of this evidence originates from studies conducted in general or mixed populations rather than exclusively rural settings. Therefore, while these findings provide important insights into the effectiveness of telemedicine, their direct applicability to rural populations remains limited and should be interpreted with caution.

In particular, differences in healthcare infrastructure, digital accessibility, availability of healthcare professionals, and patient characteristics may substantially influence both the implementation and effectiveness of telemedicine interventions in rural contexts.

Consequently, extrapolation of findings from non-rural settings to rural populations may not fully reflect real-world conditions and should be interpreted in the context of healthcare system heterogeneity.

Mobile health applications and remote monitoring systems may enable timely detection of abnormal blood pressure values, facilitate earlier intervention, and support more personalized treatment strategies.

Moreover, telemedicine interventions using SMS and interactive communication technologies have been associated with improvements in blood pressure control and treatment adherence in some studies [17]. At the same time, the extent to which these findings are applicable to rural populations remains uncertain and may depend on context-specific factors, including access to technology and patient engagement.

Importantly, the effectiveness of telemedicine may be enhanced when implemented within integrated care models involving collaboration between general practitioners and specialists. In rural settings, primary healthcare providers often serve as the first point of contact and may play an important role in coordinating long-term patient management.

Team-based care models supported by telemedicine have the potential to improve continuity of care and optimize resource utilization; however, the extent of these benefits in rural contexts may vary depending on local healthcare infrastructure and resource availability.

However, despite its potential advantages, the implementation of telemedicine in rural healthcare systems is associated with several challenges. These may include limited telecommunication infrastructure, low digital literacy among patients, lack of standardized clinical protocols, and the need for appropriate patient selection based on clinical condition [5].

Addressing these barriers is essential to ensure the effective and sustainable integration of telemedicine into routine clinical practice.

Despite the growing body of evidence supporting telemedicine in hypertension management, important gaps remain in the literature, particularly regarding its implementation in real-world rural settings. Most existing studies have been conducted in controlled environments or urban populations, where healthcare infrastructure, digital accessibility, and availability of medical resources differ substantially from rural contexts.

As a result, the applicability of these findings to rural populations remains uncertain and requires cautious interpretation. There is a need for further research to evaluate how telemedicine interventions can be effectively adapted to diverse rural healthcare systems, taking into account organizational constraints, resource limitations, and patient-specific factors.

Addressing these gaps is essential to better understand the feasibility, context-specific effectiveness, and potential limitations of telemedicine in improving hypertension outcomes in rural populations.

Given these considerations, this narrative review aims to synthesize current evidence on the role of telemedicine in the management of arterial hypertension in rural populations, with a particular focus on clinical effectiveness, organizational aspects, and implementation challenges.

## 2. Methods

This study was conducted as a narrative review aimed at synthesizing current evidence on the application of telemedicine in the management of arterial hypertension in rural populations. To enhance the transparency and comprehensiveness of the review, a structured literature search strategy was applied.

A comprehensive search was conducted in the PubMed, Scopus, and Web of Science databases to identify relevant studies published between January 2015 and December 2025. The search strategy included combinations of keywords and Medical Subject Headings (MeSH) terms, such as “hypertension,” “arterial hypertension,” “telemedicine,” “telehealth,” “remote monitoring,” “mobile health (mHealth),” “digital health,” and “rural population.” Boolean operators (AND, OR) were used to refine the search and improve retrieval accuracy.

Eligibility criteria were defined using general components commonly applied in evidence synthesis (Population, Intervention, Comparison, Outcomes, and Study design). These elements were used to guide the selection and organization of relevant studies, rather than as part of a formal systematic review framework. The population included adult patients with arterial hypertension, particularly those living in rural or resource-limited settings. The interventions of interest were telemedicine-based approaches, including remote blood pressure monitoring, teleconsultations, mobile health applications, and digital health platforms. Studies comparing telemedicine interventions with standard care or usual clinical practice were prioritized.

Eligible studies included randomized controlled trials, observational studies, and systematic reviews that evaluated the effectiveness, feasibility, or implementation of telemedicine interventions in hypertension management. The primary outcomes of interest were blood pressure control, treatment adherence, access to healthcare services, and patient satisfaction.

Studies were excluded if they were not related to hypertension, did not report clinical or patient-relevant outcomes, focused solely on technical aspects without clinical evaluation, or contained insufficient data for analysis. Non-English publications and conference abstracts without full-text availability were also excluded.

Although this study was not conducted as a formal systematic review, selected reporting principles were considered to improve the transparency and clarity of the review process. A qualitative narrative synthesis of the findings was performed.

The selection of studies was guided by general relevance criteria aligned with the objectives of the review and involved an iterative screening of titles, abstracts, and full texts. Relevant information was extracted and organized into thematic categories, focusing on study characteristics, types of telemedicine interventions, and reported outcomes.

Given the narrative nature of this review, formal risk-of-bias assessment tools were not systematically applied. Instead, the included studies were critically evaluated with regard to their methodological rigor and relevance to the research objective.

As both primary studies and evidence syntheses (systematic reviews and meta-analyses) were included, attention was paid to avoid overrepresentation of evidence. Findings from systematic reviews were interpreted at a broader level, while primary studies were used primarily for illustrative and contextual purposes rather than for quantitative aggregation.

This approach reflects a flexible and interpretive synthesis of the literature, consistent with the objectives of a narrative review, rather than a fully reproducible systematic review process.

## 3. Results

### 3.1. Telemedicine and Access to Care in Rural Populations

Telemedicine has been associated with improvements in access to healthcare services in rural and underserved regions, where geographical barriers and limited healthcare infrastructure restrict timely medical care. By reducing the need for in-person visits, telemedicine may enable patients to receive medical consultations without traveling long distances, thereby potentially reducing delays in diagnosis and treatment.

Studies conducted in rural and low-resource settings have demonstrated increased utilization of healthcare services, reduced waiting times, and improved continuity of care following the implementation of these approaches [4,5]. In addition, digital health technologies may facilitate more frequent patient–provider interactions, which are essential for chronic disease management such as hypertension.

### 3.2. Effectiveness of Telemedicine in Hypertension Management

Telemedicine may play an important role in the management of arterial hypertension, a chronic condition requiring continuous monitoring, timely treatment adjustments, and long-term follow-up. Table 1 summarizes selected studies relevant to telemedicine in hypertension management. While not all studies were conducted exclusively in rural populations, they were included due to their potential relevance to barriers and care models applicable to rural settings.

Evidence from randomized controlled trials and meta-analyses suggests that telemedicine interventions may be associated with improvements in clinical outcomes compared to standard care. However, given that a substantial proportion of this evidence originates from general or mixed populations, the applicability of these findings to rural settings remains limited and should be interpreted with caution.

Remote blood pressure monitoring, mobile health applications, and teleconsultations may contribute to improvements in blood pressure control, increased adherence to antihypertensive therapy, and enhanced patient engagement [9,10,11,12,13,14,15,16].

Several studies have reported reductions in both systolic and diastolic blood pressure among patients receiving telemedicine-based care. Moreover, digital interventions may enable earlier identification of uncontrolled hypertension and facilitate timely modification of treatment strategies.

In addition to potential improvements in blood pressure control, several studies have explored the role of telemedicine in supporting long-term disease management through continuous monitoring and feedback mechanisms. These approaches may contribute to sustained treatment adherence and improved self-management behaviors among patients.

Furthermore, the integration of telemedicine into routine care has been associated with enhanced patient engagement and more individualized treatment strategies, particularly when combined with regular follow-up and provider support. However, given the heterogeneity of study designs and populations, as well as the limited representation of exclusively rural settings, the magnitude and consistency of these effects remain uncertain and may vary depending on the type of intervention, patient population, and healthcare context.

### 3.3. Barriers to Implementation in Rural Settings

Despite evidence suggesting the potential effectiveness of telemedicine, its implementation in rural healthcare systems is associated with multiple barriers. One of the primary challenges is inadequate telecommunication infrastructure, including limited internet connectivity and limited access to digital devices. These factors restrict the scalability of telemedicine programs.

Additionally, low levels of digital literacy among patients and healthcare providers may reduce the effectiveness of telemedicine interventions. Organizational challenges, such as the absence of standardized clinical protocols and insufficient integration into routine healthcare practice, further complicate implementation [4,5].

Importantly, the shortage of healthcare professionals, particularly physicians, remains a critical issue in rural regions. This limitation affects not only the availability of traditional healthcare services but also the sustainability and expansion of telemedicine programs.

### 3.4. Telemedicine Models in Rural Healthcare

Various telemedicine models have been developed to improve hypertension management. Integrated care approaches have been associated with potential improvements. These models emphasize collaboration between primary healthcare providers and specialists, ensuring continuity and coordination of care.

A potential care model for rural settings may include an initial in-person assessment at the primary care level, followed by remote monitoring and teleconsultations using widely available digital platforms. Such an approach may facilitate regular follow-up, earlier identification of clinical changes, and more timely intervention.

Similar approaches have been explored in individual studies, including our study conducted in rural Kazakhstan [21], which suggested the feasibility of this model in a specific context. However, the generalizability of these findings remains limited and requires further validation in diverse rural healthcare settings.

In such models, general practitioners may play a central coordinating role, including coordination of patient management, monitoring clinical status, and determining the appropriateness of telemedicine use. Importantly, patient selection based on clinical condition is essential, as telemedicine may not be suitable for all cases. Therefore, the development of standardized clinical protocols and clear eligibility criteria is crucial for safe and effective implementation.

## 4. Discussion

The findings of this review suggest that telemedicine may represent a potentially useful approach to supporting hypertension management in rural populations. Evidence from previous systematic reviews and meta-analyses indicates that telemedicine interventions may be associated with improvements in blood pressure control, treatment adherence, and access to healthcare services compared to standard care [10,11,12,13,14,15,16,19,21]. However, it should be noted that a substantial proportion of this evidence originates from studies conducted in general or mixed populations, and its direct applicability to rural settings remains limited and should be interpreted with caution.

One of the potential advantages of telemedicine is its potential to address geographical barriers and disparities in healthcare access. In rural regions, where patients often face long travel distances and limited availability of healthcare facilities, telemedicine may provide an alternative approach to more continuous and accessible care. Previous studies have reported that telemedicine interventions are associated with increased healthcare utilization, improved follow-up rates, and enhanced patient satisfaction [4,5]. These outcomes may be particularly relevant for chronic disease management, including arterial hypertension, which requires regular monitoring and long-term adherence to treatment.

Despite these potential benefits, the implementation of telemedicine in rural healthcare systems remains complex and context-dependent. Organizational integration into routine clinical practice remains a significant challenge. The lack of standardized workflows, limited digital infrastructure, and insufficient institutional support may hinder the broader adoption of telemedicine programs [4,5,6]. In addition, shortages of healthcare professionals, particularly physicians, remain an important limiting factor in many rural settings.

The role of general practitioners (GPs) in the management of hypertension in rural populations appears to be relevant, particularly as primary points of contact within the healthcare system. In some healthcare settings, GPs may contribute to care coordination, although the organization of care models may vary across regions and healthcare systems. Telemedicine may support such models by facilitating communication and coordination between different levels of care.

Importantly, telemedicine should be applied with careful consideration of patient characteristics. Patient selection based on clinical condition, disease severity, and the presence of comorbidities is likely essential to ensure safety and effectiveness. Patients with unstable conditions or high-risk profiles may still require in-person evaluation and closer monitoring. Therefore, the development of standardized clinical protocols and clear eligibility criteria may be beneficial for the appropriate use of telemedicine in hypertension management.

From a public health perspective, arterial hypertension remains a major chronic condition requiring continuous monitoring and long-term management. Early diagnosis, timely initiation of treatment, and sustained blood pressure control are widely recognized as important factors in reducing the risk of cardiovascular complications, disability, and premature mortality [1,2]. Improved hypertension management may also contribute to reducing the overall burden on healthcare systems.

In addition to clinical outcomes, the potential economic and organizational impact of telemedicine should also be considered. Several studies suggest that telemedicine may contribute to more efficient use of healthcare resources by reducing unnecessary hospital visits, optimizing follow-up care, and improving time management for both patients and healthcare providers. In rural settings, where resources are often limited, such approaches may be particularly valuable in supporting healthcare system sustainability. Further research is needed to assess the cost-effectiveness and long-term economic implications of telemedicine interventions across different healthcare contexts.

Beyond these considerations, an important conceptual aspect relates to the understanding of rurality beyond geographic location. Rural populations often experience differences in care complexity and clinical trajectories, including delayed diagnosis, fragmented follow-up, and a higher burden of uncontrolled chronic conditions. These factors may influence both the implementation and effectiveness of telemedicine interventions. In this context, telemedicine should not be viewed as a uniform solution but rather as a context-sensitive strategy that requires adaptation to the specific clinical and organizational characteristics of rural healthcare systems.

Overall, telemedicine may represent a potentially useful strategy for strengthening healthcare delivery in rural areas. However, its successful implementation likely depends on multiple contextual factors, including infrastructure development, training of healthcare professionals, patient digital literacy, and integration into existing healthcare systems. Therefore, the generalizability of these findings may vary across different rural settings and healthcare environments.

### Limitations

This review has several limitations. First, as a narrative review, it does not provide pooled quantitative estimates of effect. Second, only English-language publications were included. Third, heterogeneity in study design and telemedicine interventions limits direct comparison across studies.

## 5. Conclusions

Telemedicine may represent a potentially useful approach to improving hypertension management in rural populations. It may enhance access to healthcare services, support continuous monitoring, and facilitate treatment adherence. However, the strength of the available evidence varies, and a considerable proportion of studies are not exclusively focused on rural settings.

The implementation of telemedicine in hypertension care should be interpreted within the context of specific healthcare systems, infrastructure, and patient populations. While primary care providers, including general practitioners, may play an important role in care delivery and coordination in certain settings, these roles may differ across regions and healthcare models.

Future research should focus on generating more context-specific evidence, particularly in rural populations, as well as evaluating the long-term clinical and economic impact of telemedicine interventions.

## Figures and Tables

**Table 1:** Selected studies on telemedicine interventions in hypertension management with relevance to rural settings.

Author (Year)	Country	Study Design	Population	Intervention	Key Findings
Sakima et al. (2025) [9]	Japan	Systematic review and meta-analysis	Adults with hypertension	Digital health interventions	Reported reductions in blood pressure; overall evidence suggests potential effectiveness, though heterogeneity exists across studies.
Li et al. (2020) [18]	Global	Meta-analysis	Adults with hypertension	mHealth interventions	Associated with improvements in blood pressure control and self-management in several studies.
Zangger et al. (2023) [19]	Global	Systematic review	Patients with chronic conditions	Digital health interventions	Reported improvements in selected clinical outcomes and physical activity levels.
Beger et al. (2025) [10]	Germany	Pragmatic randomized controlled trial	Primary care patients	Digital blood pressure coaching	No significant difference in blood pressure reduction compared to standard care; improved adherence to home blood pressure monitoring.
Liu et al. (2020) [11]	Canada	Randomized controlled trial	Patients with hypertension	E-counseling intervention	Associated with improvements in lifestyle behaviors and blood pressure outcomes.
Gong et al. (2020) [15]	China	Multicenter randomized controlled trial	Patients with hypertension	Mobile health application	Reported reductions in blood pressure compared to standard care.
Or et al. (2020) [14]	China	Randomized controlled trial	Patients with diabetes and hypertension	Technology- supported self-care	Associated with improvements in treatment adherence and blood pressure control.
Dorsch et al. (2020) [16]	USA	Randomized controlled trial	Adults with hypertension	Mobile application (LowSalt4Life)	Reported reductions in sodium intake and improvements in blood pressure outcomes.
Cho et al. (2020) [17]	South Korea	Randomized controlled trial	Community population	Smartphone-based lifestyle coaching	Improved cardiovascular risk factors
Schroeder et al. (2020) [20]	USA	Randomized controlled trial	Patients with hypertension	SMS and interactive voice response intervention	Associated with improvements in blood pressure control and adherence.
Jahan et al. (2020) [4]	Bangladesh	Randomized controlled trial	Rural population	mHealth intervention	Associated with increased awareness and healthcare utilization in rural settings.
Bilmakhanbetova et al. (2026) [21]	Kazakhstan	Two-phase study	Rural patients with hypertension	Telemedicine-based care model	Reported high patient satisfaction and feasibility, with increased trust in telemedicine-based care in a rural setting.

Note: While not all included studies were conducted exclusively in rural populations or focused solely on hypertension, they were selected based on their relevance to telemedicine implementation and care models applicable to rural settings. Their findings were interpreted with caution, considering differences in healthcare infrastructure, access, and patient characteristics.

## Data Availability

Not applicable, as this study is a narrative review based on previously published data.

## Abbreviations

BP	blood pressure
mHealth	mobile health
GP	general practitioner
